# Establishment of a Stacking Machine Learning Model Predicting Cardiac Phenotype in Ectopia Lentis Patients Based on Genotype and Ocular Phenotype

**DOI:** 10.7150/ijms.109657

**Published:** 2025-07-28

**Authors:** Linghao Song, Ao Miao, Xinyue Wang, Yan Liu, Xin Shen, Zexu Chen, Wannan Jia, Yalei Wang, Xinyao Chen, Tianhui Chen, Yongxiang Jiang

**Affiliations:** 1 Eye Institute and Department of Ophthalmology, Eye & ENT Hospital, Fudan University, Shanghai, 200031, China.; 2 Key laboratory of Myopia and Related Eye Diseases, NHC; Key laboratory of Myopia and Related Eye Diseases, Chinese Academy of Medical Sciences, Shanghai, 200031, China.; 3 Shanghai Key Laboratory of Visual Impairment and Restoration, Shanghai, 200031, China.

**Keywords:** Machine Learning, Phenotype, Genotype, Ectopia Lentis

## Abstract

**Purpose:** To establish a stacking machine learning model for cardiac phenotype prediction in ectopia lentis (EL) patients on the basis of their genotype and ocular phenotype.

**Methods:** We enrolled 151 patients with congenital EL and divided them into three groups according to their echocardiograph (normal group, reflux group, and organic lesion group). All the subjects underwent genetic screening and an up-to-1-year ophthalmic and cardiac follow-up. Patients were randomly divided into training set and validation set in a 3:1 ratio. Six statistically significant parameters based on one-way ANOVA and regression analysis were fed into nine basic algorithms for diagnostic training.

**Results:** Among the three groups, intergroup differences in axial length and central corneal thickness were identified. In genotypes, patients with cysteine-eliminating dominant negative and homozygous deficiency mutations were predisposed to cardiac abnormalities. In addition, the corneal radius of curvature and the mutation domain were also included in the experimental dataset. In the validation set, the diagnostic model achieved a comprehensive accuracy of 75% for predicting cardiac phenotype.

**Conclusion:** We established a reliable machine-learning model which predicts cardiac phenotype using genotype and ocular phenotype in EL patients. This model possibly facilitates effective diagnosis of Marfan syndrome.

## Introduction

Discovered at the end of the 19th century 1, 2, Marfan syndrome was cognized as an autosomal dominant connective tissue disease that can involve cardiovascular, musculoskeletal, ocular and other systems 3-5. Mutations in FBN1 gene, encoding fibrillin-1, can be detected in nearly 90% of Marfan patients 6-8. Studies have reported that the probability of sudden death due to disease-related events is around 25%-30% 9, 10, and the average life expectancy was only 32 without prompt intervention 10, 11. Therefore, the timely diagnosis and treatment of Marfan syndrome should attract the attention of both doctors and patients.

Over 60% potential Marfan patients are suspected because of EL in childhood 12, while acute aortic dissection due to aortic root dilatation after adulthood is their most common life threat 13. In previous studies, we established a diagnostic model based on genotype and ocular phenotype, which increased the comprehensive diagnosis rate from 19.43% to 40.57% 14, 15. However, 62.83% patients still cannot rule out the possibility of simple lens dislocation syndrome due to the lack of clear diagnosis of heart disease in children in the growing stage 14, 16, 17. Even in the latest Ghent-2 criteria, juvenile patients can only be classified as "potential Marfan" in which echocardiography is recommended until 20^17^. This practice leads to another question, that is whether echocardiography is equally necessary for all patients with EL.

Many have paid attention to the correlation between genotype and phenotype in patients with FBN1 mutations in previous studies. Stengl et al. found that the homozygous deficiency (HI) mutation and cysteine-eliminating (-Cys) dominant negative (DN) mutation in FBN1 gene had a significantly higher incidence of aortic involvement than other mutation types, which provided a basis for the classification of FBN1 gene mutations with high heterogeneity 18. Our studies found that patients with DN (-Cys) mutation have longer axial length 19 while those with HI and neonatal region mutations have thinner central corneal thickness 16. However, there hasn't been a model that can fully consider the association between FBN1 genotype and cardiac and ocular phenotypes, through which possible cardiac problems can be predicted.

Hence, we carried out this study, collecting the genetic and echocardiographic reports of patients undergoing EL surgery, exploring the association between genotype, ocular phenotype, and cardiac phenotype based on cardiac classification, and developing a prediction program for cardiac conditions through machine learning. We finally realized the aim of I: achieving an accuracy of 75% in predicting the cardiac phenotype of EL patients, II: proposing possible explanations for some cases with short axial length (AL) 20, III: predicting the cardiac outcome in patients with EL through a new three-category system (normal, regurgitation and organic type).

## Methods

A total of 151 patients with congenital EL were included in this study. The data of genotype, cardiac and binocular ocular parameters were collected. These patients underwent EL surgery between July 2016 and July 2023 at Eye & ENT Hospital of Fudan University. The study was conducted in strict accordance with the principles of the Declaration of Helsinki. In addition, it was approved by the Human Research Ethics Committee of the Eye & ENT Hospital of Fudan University.

### Inclusion, exclusion and grouping criteria

From July 2016 to July 2023, a total of 406 patients with congenital EL were confirmed FBN1 mutation by gene detections. Except patients with (1) complete dislocation into the anterior chamber or vitreous; (2) history of ocular trauma or surgery prior to dislocation, the remaining 393 patients were followed up. A total of 151 patients who had long-term cardiac examination after operation and willing to provide echocardiography reports were enrolled in this study.

**Figure 1:**
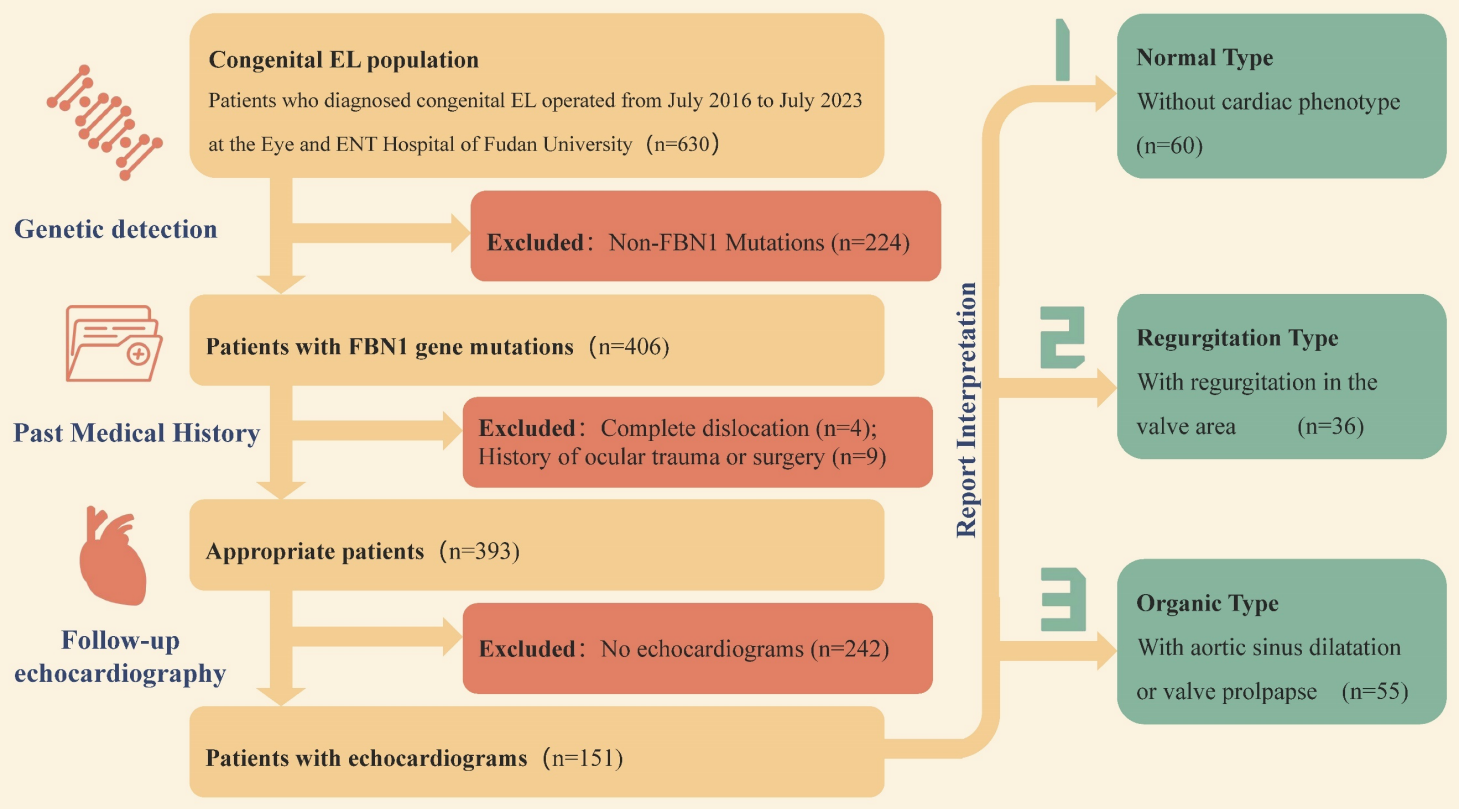
**Patient inclusion and exclusion and grouping criteria**.

According to the results of echocardiography, the patients were divided into three groups with the help of cardiologists. Finally, 60 patients with normal cardiac phenotype, 36 patients with valve regurgitation, and 55 patients with organic lesions were determined. The specific inclusion and exclusion process and grouping process are shown in Figure 1.

### Ophthalmologic and systemic examinations

All enrolled patients underwent comprehensive eye examination. Slit-lamp examination was conducted with mydriasis. We defined the EL as the visible lens edge or lens tremor under mydriatic slit-lamp biomicroscopy inspections. Preoperative ocular parameters were measured by partial coherence interferometry (IOLMaster 700; Carl Zeiss Meditec AG). All examinations were performed by the same blinded experienced optometrist. Best corrected visual acuity (BCVA) and spherical equivalent (SE) for each eye as well as ocular biometric parameters including AL, corneal radius of curvature (CCR), central corneal thickness (CCT), corneal astigmatism, lens thickness (LT), and white to white (WTW) were analyzed separately. Z-scores of AL, CCR and WTW are calculated by the formula: Z-score = (measured parameter - normative parameter)/normative standard deviation (SD), which can standardize the influence of age difference on the parameters, and evaluate their levels in different age groups.

The cardiac phenotype of the patients was determined through echocardiographic examination (Aloka Arietta 60 ultrasound machine) at a tertiary general hospital and were reported by a dedicated cardiologist. During follow-up, the patients' most recent postoperative echocardiographic findings were interpreted to represent their true cardiac phenotype.

### Genetic screening and mutation classification

A customized congenital EL panel consisting of 41 genes was generated from genes identified in previous genetic screening of the Chinese Marfan cohort or genes reported to be associated with EL or Marfan in studies 14, 21, 22. A DNA library from peripheral blood was used for panel based next generation sequencing (NGS) on an Illumina Novaseq 6000 platform (Illumina Inc., San Diego, CA, USA) 23. The reference sequence of FBN1 transcript was NM_000138. Ensembl Variant Effect Predictor 105, an integrated network tool, was used Computer analysis (http://uswest.ensembl.org/info/docs/tools/vep/index.html), Including splice site prediction (SpliceAI), allele frequency annotation (gnomAD), and missense prediction (MutationTaster, PolyPhen, and SIFT). Candidate variants were validated by Sanger sequencing. The SALSA MLPA Probemix kit (# p0665 -C1/ p0666 -C1; MRC Holland) for patients in whom a causative variant could not be detected after data reanalysis. Genotype-phenotype cosegregation analysis was performed on family members, and all variants were assessed for pathogenicity according to the American College of Medical Genetics and Genomics guidelines 24.

FBN1 variants were first divided into two groups according to mutation effect: the dominant negative (DN) group, which consisted of missense variants and group codon deletions or insertions, and the haploinsufficiency (HI) group, which included frameshift variants, nonsense mutations, splicing variants, and base deletions or duplications. In the DN group, the DN (-Cys) variant, which was prone to aortic involvement, was divided into high-risk group with HI variant according to previous reports 18, and the differences of cardiac phenotype were compared with DN (others) group*.*

### Statistical analysis

All statistical analyses were performed in SPSS 20.0 (IBM Corp., Armonk, NY, USA). The mean and standard deviation were used to represent the central tendency and statistical dispersion of the measured data, while the dichotomous data were presented in a four-grid table form. The Shapiro-Wilk test was used to test sample normality. One-way ANOVA was used to confirm the statistical significance of the differences of nominal parameters among the normal, reflux, and organic patients, and the chi-square test or Fisher's exact test was used to compare the categorical variables. Pearson correlation coefficient test was used to verify the consistency of ocular parameters in the same patient, so that the one-to-one correspondence between ocular phenotype and cardiac phenotype was established. A two-sided *P* value of less than 0.05 was considered to indicate statistical significance.

### Machine learning

Enrolled patients were randomly assigned to the training set and the validation set in a ratio of 3 to 1. The data of the validation set were retained exclusively and not involved in the model building. 6 variables were input into 9 base algorithms as primary learner, then the results predicted by the primary learner on the training set are taken as new features, and together with the features of the original training set, to form the training set of the secondary learner. Through the ensembled classification of the secondary learner, a predicted cadiovascular outcome was produced. The 9 base machine learning algorithms include Multinomial Logit Model (multinom), Decision Tree (DT), Real-Time Semantic Segmentation (ENet), K-Nearest Neighbor (KNN), Light Gradient Boosting Machine (Lightgbm), Random Forest (RF), EXtreme Gradient Boosting (XGBOOST), Support Vector Machine (SVM) and Multilayer Perceptron (MLP). We hope that by considering different basic algorithms, the final model will be stable and reliable, while maximizing the information benefit of the data. For the selection of hyperparameter space, we search the hyperparameters as comprehensively as possible, and finally give the range of hyperparameters in a state that makes the training time and training accuracy relatively balanced.

**Figure 2:**
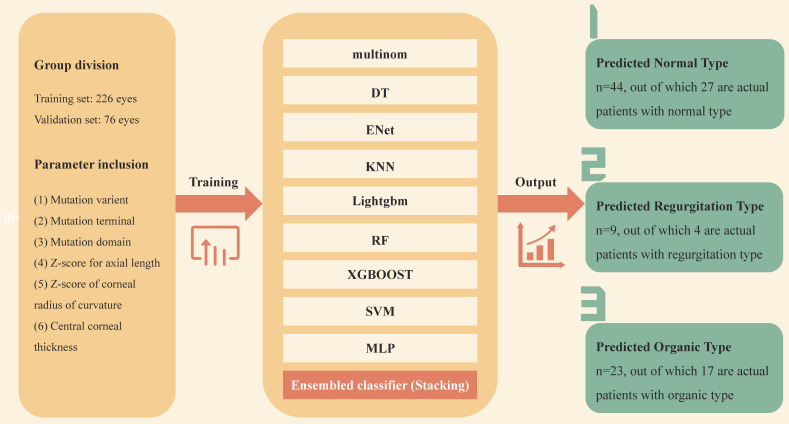
**The establishment and output of stacking ensemble machine learning model.** Multinom: Multinomial Logit Model; DT: Decision Tree; ENet: Real-Time Semantic Segmentation; KNN: K-Nearest Neighbo;Lightgbm: Light Gradient Boosting Machine; RF: Random Forest; XGBOOST: EXtreme Gradient Boosting; SVM: Support Vector Machine; MLP: Multilayer Perceptron.

Univariate characteristics analysis was performed on the predictors to measure the importance of genotype and ocular phenotype in predicting cardiac outcomes. Then, according to the score and *P* value, the output vectors of the basic learner were integrated into the input meta-learner Lasso regression model to perform stacking ensemble machine learning (SEML) on the prediction results, and a multi-modal stacked ensemble dataset was formed. The establishment and output of the machine learning model is depicted in Figure 2. For the samples of three classification outcome variables, we adopted the "One-vs-Rest" strategy and plotted the corresponding receiver-operating-characteristic (ROC) curve when each outcome classification was taken as a positive class so as to test the predictive performance. The hyperparameter penalty of Lasso is determined according to the aim of maximizing the area under the curve (AUC) of ROC (Figure 4I).

## Results

### Differences in ocular phenotypes among patients with different cardiac phenotypes

The demographic information and ocular parameters of the patients are shown in Table 1. Among all the ocular parameters, the Z-scores of AL and CCT were statistically significant, with the P value among groups under 0.001. While the Z-score of AL was quite special -- the organic group was the group with the longest AL, but the regurgitation type group had significantly shorter AL than the other two groups. The CCT showed a stepwise change trend: the normal group was the thickest, followed by the regurgitation group, and organic type group was the thinnest. There were no significant differences in other ocular parameters among the three groups (P > 0.05).

**Figure 3:**
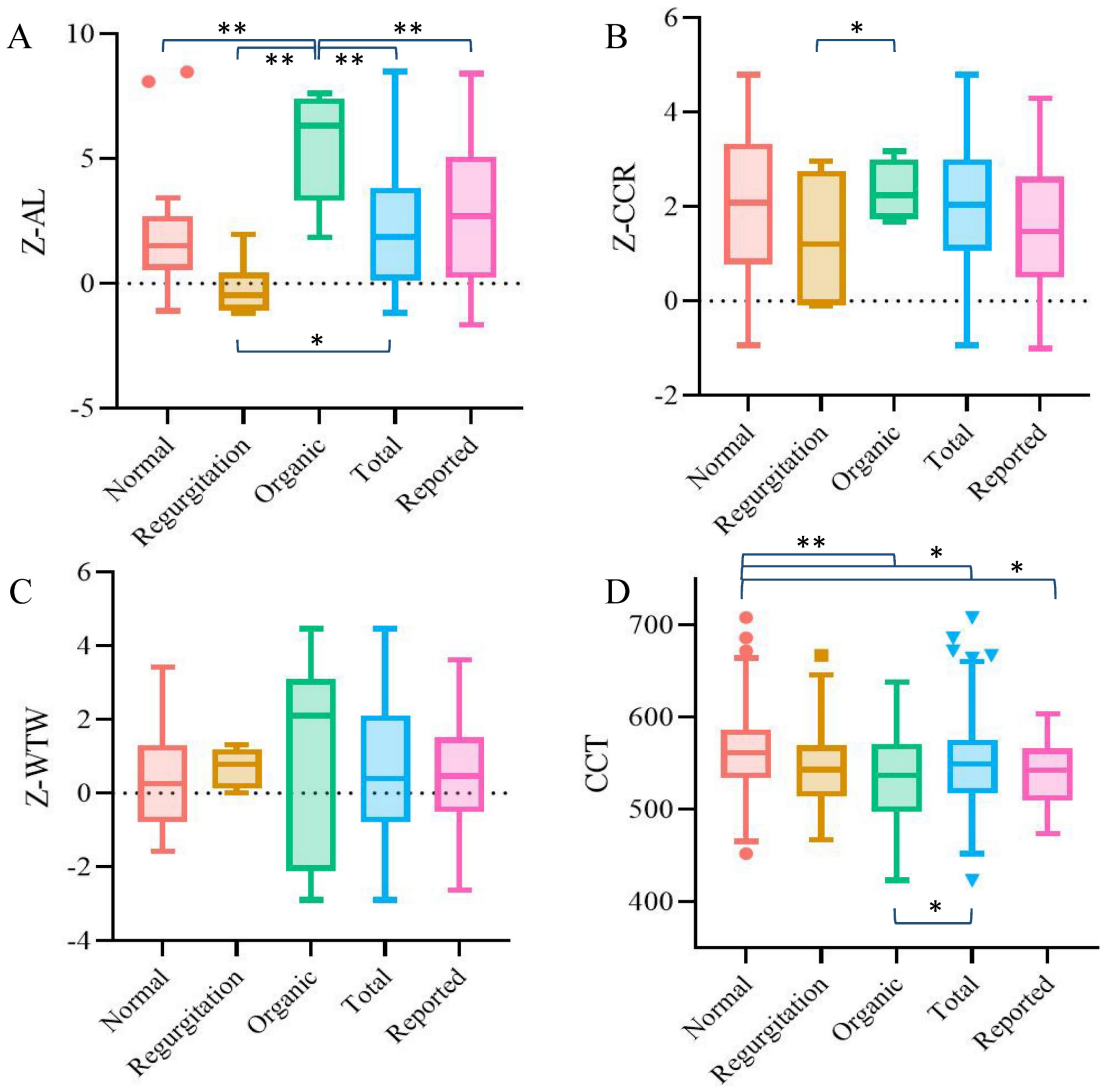
**Agreement analysis between the ocular parameters of each group in this study and the data reported in the previous literature.** A. Z-AL; B. Z-CCR; C. Z-WTW; D. CCT. Results that were statistically significant (*P< 0.05, **P<0.001) were marked accordingly on the graphs.

In order to verify the reliability of the data obtained in this study, we compared the ocular parameters of each group and the whole cohort with the previous literature. Since the Z-score algorithm of ocular parameters in Marfan patients was differentiated by age, we selected 6-year-old children in our study group for comparison 20. The results showed that for the total cohort, the AL, CCR and WTW of the enrolled patients in this study were not significantly different from those in previous reports, while the between-group difference was only found in AL. The mean CCT of the patients in this study was also similar to the previous conclusion 25, but the thicker CCT of the patients with normal heart was particularly significant compared with the other groups (Figure 3).

### Genotype differences in patients with different cardiac phenotypes

The results of comparison of genotype characteristics in patients with the three classes of cardiac phenotypes are shown in Table 2. Patients with DN (-Cys) or HI mutations were more likely to have cardiac phenotypes than those with DN (Others) mutations. According to the structure of the FBN1 gene, it was divided into the N terminal, the middle region and the C terminal, in which the propensity of mutation sites in the three types of patients was obvious. Most mutations in the C terminal of the FBN1 gene led to the normal cardiac type, while the middle region mutation was the main cause for the organic shift. The proportion of C terminal mutation in regurgitation type (44%) was much higher than that in the other two groups (23% and 16%, respectively). However, if the view was further refined at the domain level, because most of the mutations occurred in the cb EGF-like domain, which related to the structural composition of FBN1 gene 26, there was no difference in the mutated domain among the three groups of patients.

### Performance evaluation of SEML model

**Figure 4:**
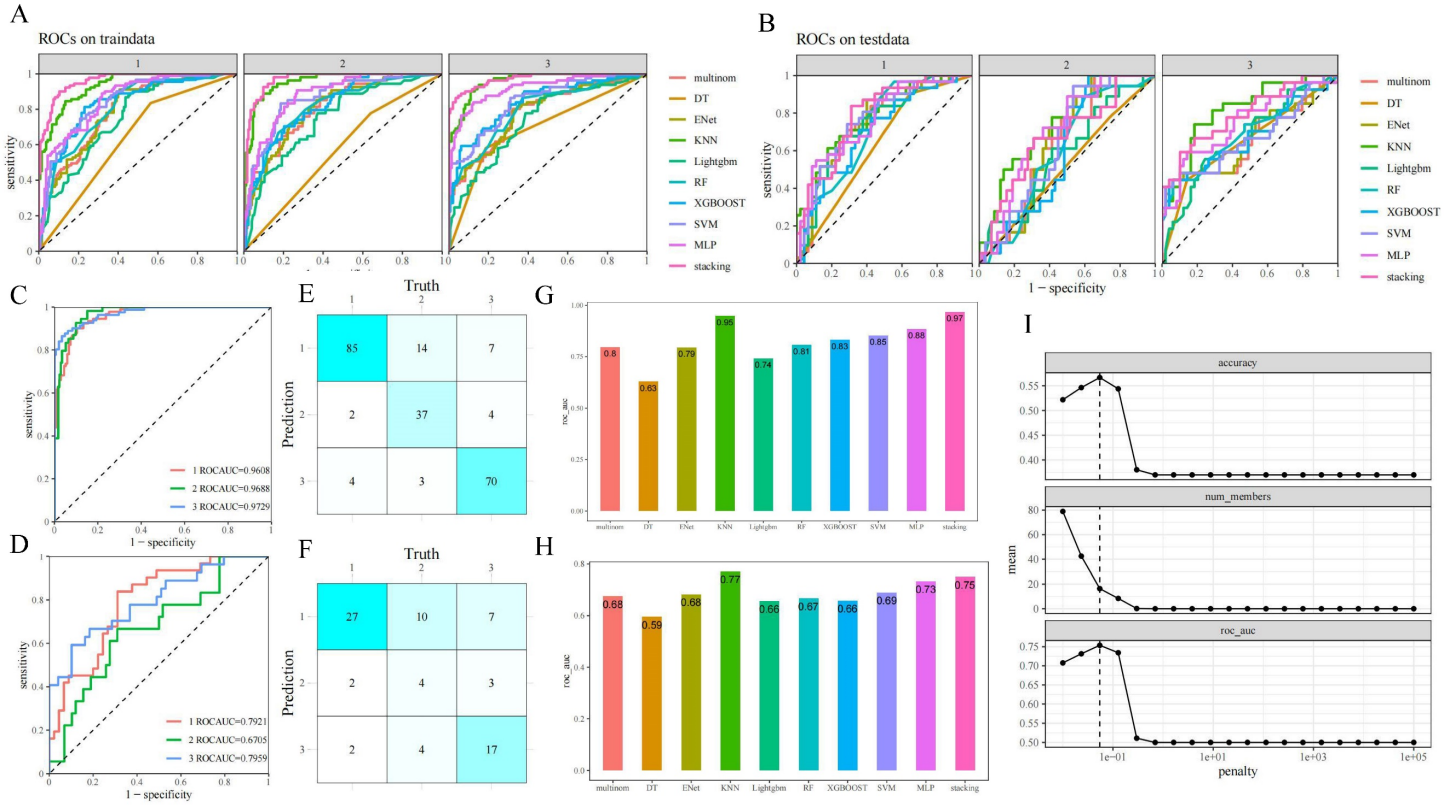
**Performance evaluation of machine learning.** A,B. ROC-AUC of the base algorithm as well as SEML model for the three sets of cardiac phenotypic outcomes in training set (A) and validation set (B); C,D. Clear presentation for ROC-AUC of the SEML model for the three sets of cardiac phenotypic outcomesin training set (C) and validation set (D); E,F. Nine-square grid of the correspondence between machine prediction and truth values in training set (E) and validation set (F); G,H. Integrated ROC-AUC of the base algorithm (G) and SEML model (H); I. Hyperparameter penalty confirmation for the meta-learner Lasso regression of SEML model.

The consistency test revealed that there were no significant differences in the basic demographic and biological parameters of the included populations in the training set and the validation set (Supplementary Table 1). The results of the precise segmentation of the respective diagnostic performance of the nine-base algorithms as well as SEML in the training and validation set by cardiac phenotypes are shown in Figure 4 (A, B) and Supplementary Figure 1 (A, B). The performance of stacking ensemble learning can be more intuitively represented in Figure 4 (C-F) and Supplementary Figure 1 (C, D). Among the three cardiac phenotypes, the SEML performed relatively well in distinguishing between the organic and normal cardiac phenotypes, which are broadly understood as Marfan and non-Marfan patients, with ROC-AUC of 0.7959 and 0.7921 in the validation set, respectively. In addition, the area under the precision versus recall curve (PR-AUC) of them also reached 0.7566 and 0.7201. However, there was a slight decrease in the prediction accuracy for patients of regurgitation type, with ROC-AUC of 0.6705 and PR-AUC of 0.3891 under the same criteria, which indicates that there was a tendency to overpredict or underpredict the severity in regurgitation patients. The confidence intervals for both curves are shown in Supplementary Figure 1 (E, F).

The combined diagnostic yield obtained by integrating the three cardiac phenotypes is shown in Figure 4 (G, H) and Supplementary Figure 1(G, H). Among the nine-base algorithm, KNN showed the best prediction performance with an accuracy, precision and recall of 0.81 in the training set, aside with a final ROC-AUC of 0.95. After SEML deep learning, the ROC-AUC of the training set reached the highest value of 0.97. While in the testing set, the ROC-AUC of SEML model integrated with the 9 basic algorithms reached 0.75. The accuracy, precision and recall were 0.63,0.6 and 0.57, respectively.

## Discussion

Marfan syndrome often involves cardiovascular, musculoskeletal and ocular systems, among which EL is usually the early onset manifestation, while cardiovascular events are fatal threats 27. According to the Ghent-2 criteria, the diagnosis of cardiovascular changes in Marfan syndrome cannot be define until the age of 20 years 17, but the occurrence of EL is as early as 3-4 years of age 28, 29. Due to the significant time difference in the onset of Marfan's cardiac and ocular phenotype, there is still a lack of methods to make a definite diagnosis of juvenile patients, and children can only be asked to follow up continuously. For both doctors and patients, the potential risks caused by a long follow-up period cannot be estimated. Therefore, whom should more attention in cardiac follow-up be paid to is an urgent clinical problem to be solved.

Given that Marfan syndrome is a rare disease, it is hard to collect such sample size as ours, and it's also difficult to complete a risk assessment based solely on a clinician's personal experience. Machine learning can integrate clinical clues that are easily overlooked to achieve early identification and diagnosis of diseases 30. Nowadays, with the advancement of technology, even for small samples of rare disease models, machine learning can obtain acceptable robust models through data enhancement 31. On this basis, we hope to assess the risk of later cardiac disorder in adolescent EL patients by integrating the genotype with the cardiac and ocular phenotypes. Individually, patients with cardiac phenotype (regurgitation and organic) were significantly different from those without cardiac phenotype in terms of AL, CCT, mutation variant and mutation terminal, which is consistent with previous reports 18, 25, 32, 33. Our machine learning-based strategy provided a further and clearer reference. By integrating the patient's genetic report and ocular parameters, the patients were divided into three categories according to the cardiac phenotype, and multiple regression analysis provides a basis for us to establish a better diagnostic model. Among the three heart phenotypes, our model had the strongest discrimination power for organic type, with both ROC-AUC and PR-AUC exceeding 0.75. This is a significant improvement over the previous diagnostic yield of pediatric Marfan, which used to be only around 40% 14.

The unique feature of our diagnostic model is that the cardiac outcome of potential Marfan patients is divided into three categories. Among them, the regurgitation type is defined as the diagnosis of mild or greater regurgitation according to the ultrasonic diagnostic criteria of valve regurgitation 34, 35. These patients have a relatively lower probability of serious cardiac accidents than organic type, but there are risks of arrhythmia, palpitations and other cardiology diseases. This classification can further subdivide Marfan syndrome according to the severity of cardiac lesions. According to the results of this study, there are still some differences between the genotype and ocular phenotype of patients with organic and regurgitation lesions, which may partly explain the existence of some special cases of Marfan in the past clinical reports.

Long AL is a common ocular phenotype in Marfan patients 36. However, there are still some studies reported that nearly 30% of Marfan patients have short AL 20, 37. Under our three-classification model, the origin of the difference between long and short AL seems to be gradually clear: those patients with pure regurgitation type depicts significantly shorter AL than the organic type or even those without cardiac phenotype. In this study, the proportion of patients with regurgitation type of echocardiography was 23.8%, which suggests that there is a correlation between the cardiac and ocular phenotypes in Marfan patients.

Previous studies on various congenital heart diseases have confirmed that the pathways of FBN1 gene mutations leading to changes in cardiac structure are multi-faceted. On the one hand, as a component of the aorta, FBN1 mutation leads to decreased elastin activity, damaged structure, and cystic necrosis caused by fiber rupture in the middle of the aorta, which contributes to the occurrence of aneurysms 38, 39. On the other hand, due to the high homology between FBN1 and LTBP, FBN1 deficiency leads to increased TGF-β level, activation of the TGF-β pathway, and dysregulation of signal transduction 40. TGF-β family signaling is involved in the endothelial-mesenchymal transition (EndMT) of vascular and lymphatic cells. During embryonic development, mesenchymal cells migrate to the central glia and promote the formation of heart valves 41, 42. However, a large number of examples have shown that excessive activation of the TGF-β pathway can lead to pathological activation of EndMT, excessive loss of microvascular endothelium, and promote the influx of inflammatory cells such as macrophages and T cells, which lead to valve remodeling and thickening, mechanical properties changes, and even cardiac fibrosis 43, 44. This is also consistent with the cardiac anatomy of patients with congenital valvular regurgitation 45. In the eye, elevated TGF-β signaling has also been shown to be associated with inhibition of ocular vascular development 46. Therefore, here we propose two hypotheses for the mechanism of the short AL in some Marfan patients: 1. Direct effect: certain FBN1 gene mutation activates TGF-β signaling pathway in patients with regurgitation lesions, leading to loss of ocular microvascular endothelial growth, inhibition of development, affecting eyeball development, and resistance to axial growth; 2. Indirect effect: Due to the presence of valvular regurgitation, the blood pumping of the heart is relatively reduced, the nutrients supplied to the ocular capillaries are reduced, and the eyeball development is slowed down.

There have been many studies on the association between Marfan genotype and phenotype, but few have focused on the association and differences between cardiac and ocular phenotypes among different patients. In our three-category case, the significant contribution of high-risk mutation variants to the incidence of both cardiac phenotypes was not different from that seen in the general case 18, 47. However, by studying the mutation terminal, the uniqueness of different types can be observed. N-terminal mutations are the most common mutation sites in gene segments, and more than half of the mutations occur in this region. Meanwhile, the N-terminal of FBN1 is also the most common site in patients with normal heart type, with more than 70% of normal patients having N-terminal mutations of FBN1.Mutations in the middle region were found to have the highest risk, with 60% of patients having organic heart disease, and mutations in the neonatal region (exons 24-32), a region clinically associated with high-risk Marfan 48, were found to have organic heart disease in more than 90% of patients. The lowest proportion of mutations was found in the C-terminal region, which was consistent with our previous study 49, but the highest proportion of patients with regurgitation heart disease (43%) was found in this region. In the TGF-β regulatory region of exon 44-49 49, the proportion of reflux heart disease was 57.14% (4/7), which was also consistent with the possibility of the association between TGF-β signaling pathway and valvular regurgitation discussed above. Therefore, we believe that our three-category prediction model for potential Marfan outcomes is well supported.

However, our study still has some limitations: 1. Our data size was limited. Only 151 people were finally included in the cohort after screening and follow-up. This sample size would lead to too few cases when conducting segmented personalized prediction. For example, only 7 patients who were to detect the TGF-β regulatory region mutation as mentioned above. Such lack of enough cases would affect the accuracy of the model. Therefore, we can only establish the model with a relatively extensive branch, and if there is more abundant sample data, more personalized and fine prediction can be achieved. The accuracy of machine-learning in predicting cardiac phenotypes might have also been further improved with a larger sample size 2. Our follow-up time of patients is limited, and the echocardiographic results of most patients in the experiment are only based on the reports closest to the current time, rather than the most realistic results of patients when they are real adults, so our study should be used only as a prediction instead of a diagnosis. 3. The generalizability of our model cannot be confirmed due to the lack of validation with external data. We hope that a broader population data can be obtained to verify our model prediction performance.

In conclusion, our model takes genotype, ocular phenotype and cardiac phenotype into consideration, and the comprehensive prediction accuracy is satisfactory. This model not only contributes to speculating patients' cardiac outcome, but also provides a new perspective and idea for us to understand and explore Marfan syndrome.

## Supplementary Material

Supplementary table.

## Figures and Tables

**Table 1:** Demographic information and ocular parameters for the three categories of patients and overall.

	Normal	Regurgitation	Organic	Total	*P*-value
Eyes	120	72	110	302	
Gender (M:F)	34:26	25:11	41:14	100:51	0.013
Age	8.18±7.71	8.47±5.07	11.80±10.80	9.57±8.64	0.003
Z-AL	2.01±2.33	0.78±2.10	2.73±3.32	1.98±2.79	<0.001
Z-CCR	1.86±1.38	1.77±1.30	2.18±1.26	1.96±1.32	0.081
Z-WTW	0.20±1.34	0.25±1.37	0.60±1.45	0.36±1.40	0.131
CCT	566.99±55.46	544.16±38.78	537.77±48.87	551.06±51.15	<0.001
Cyl	-1.76±0.82	-1.85±1.12	-1.73±1.06	-1.77±0.99	0.719
CECs	3261.98±493.10	3326.35±422.95	3239.90±418.03	3268.99±451.28	0.486
IOP	15.18±3.67	14.76±3.72	14.59±4.07	14.86±3.84	0.548
PO-1m BCVA	4.70±0.19	4.77±0.17	4.72±0.20	4.72±0.19	0.063

Z-AL: Z-score for axial length; Z-CCR: Z-score of corneal radius of curvature; Z-WTW: Z-score for white to white; CCT: central corneal thickness; Cyl: corneal astigmatism; CECs: corneal endothelial cells; IOP: intraocular pressure; PO-1m BCVA: best corrected visual acuity at 1 month after surgery

**Table 2:** Comparison of the three groups of patients and the whole population according to the mutation varient, mutation terminal and mutation domain.

		Normal	Regurgitation	Organic	Total	*P*-value
Variants	DN(-Cys)&HI	23	20	31	74	0.039
	DN(Others)	29	11	17	57	
Terminal	N-terminal	40	17	16	73	< 0.001
	Middle Region	10	7	26	43	
	C-terminal	4	7	5	16	
Domain	cb EGF-like	27	21	31	79	0.067
	EGF-like	7	6	3	16	
	TGFBP	7	1	4	12	
	Hybrid	4	1	4	9	
	LTBP-like	6	0	0	6	
