# Supplementary material for: Establishment of a Stacking Machine Learning Model Predicting Cardiac Phenotype in Ectopia Lentis Patients Based on Genotype and Ocular Phenotype
Source: Int J Med Sci. 2025 Jul 28;22(14):3501–10. doi: 10.7150/ijms.109657 (PMC12434693; doi:10.7150/ijms.109657)
Supplement: Supplementary file 1 — Supplementary table. [file ijmsv22p3501s1.pdf]

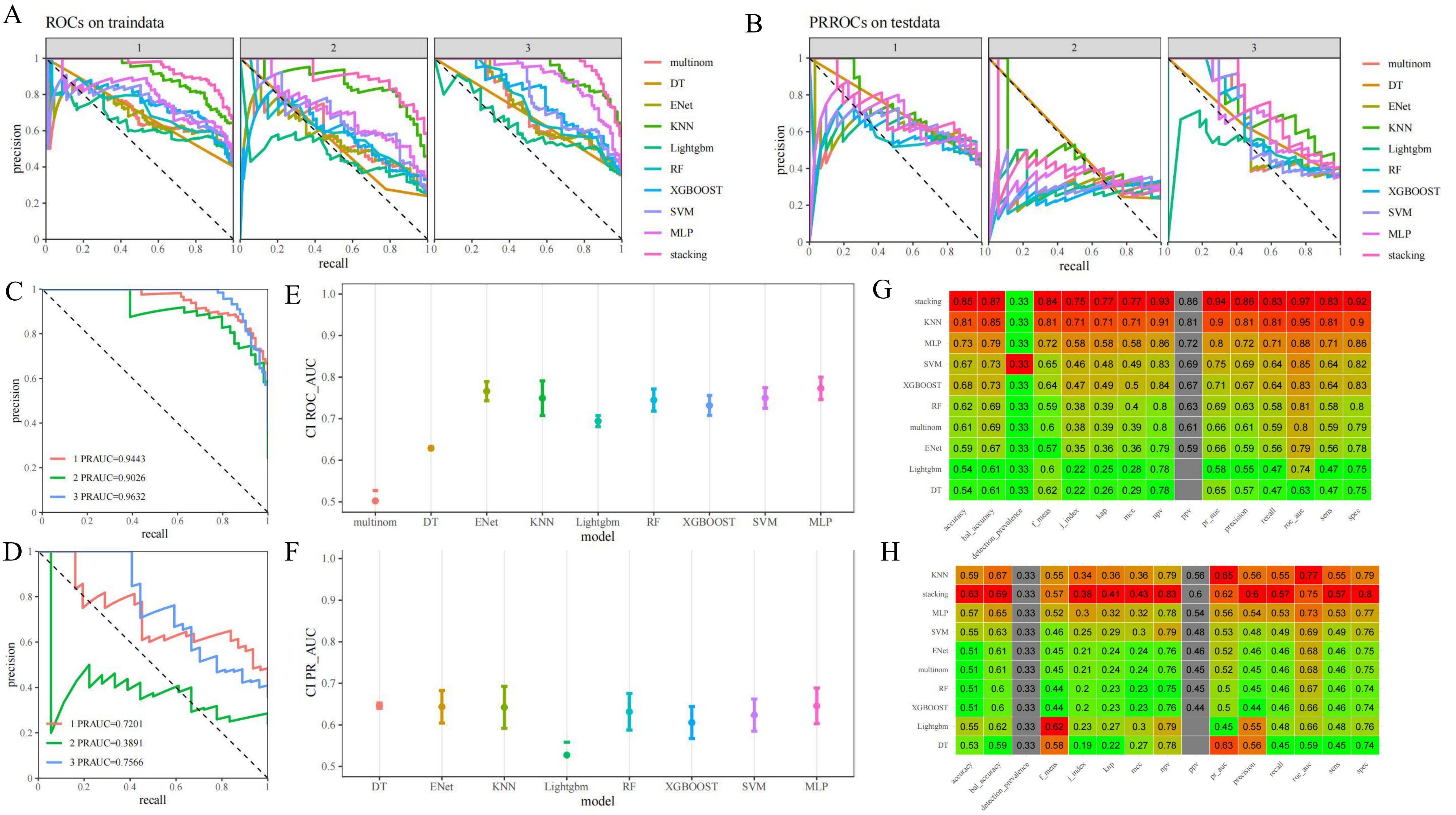
A
ROCs on traindata
B
PRROCs on testdata
precision
recall
multinom
DT
ENet
KNN
Lightgbm
RF
XGBOOST
SVM
MLP
stacking
C
1 PRAUC=0.9443
2 PRAUC=0.9026
3 PRAUC=0.9632
D
1 PRAUC=0.7201
2 PRAUC=0.3891
3 PRAUC=0.7566
E
CI ROC_AUC
model
F
CI PR_AUC
G
H
accuracy bal_accuracy detection_prevalence f_meas j_index kap mcc npv ppv pr_auc precision recall roc_auc sens spec
stacking 0.85 0.87 0.33 0.84 0.75 0.77 0.77 0.93 0.86 0.94 0.86 0.83 0.97 0.83 0.92
KNN 0.81 0.85 0.33 0.81 0.71 0.71 0.71 0.91 0.81 0.9 0.81 0.81 0.95 0.81 0.9
MLP 0.73 0.79 0.33 0.72 0.58 0.58 0.58 0.86 0.72 0.8 0.72 0.71 0.88 0.71 0.86
SVM 0.67 0.73 0.33 0.65 0.46 0.48 0.49 0.83 0.69 0.75 0.69 0.64 0.85 0.64 0.82
XGBOOST 0.68 0.73 0.33 0.64 0.47 0.49 0.5 0.84 0.67 0.71 0.67 0.64 0.83 0.64 0.83
RF 0.62 0.69 0.33 0.59 0.38 0.39 0.4 0.8 0.63 0.69 0.63 0.58 0.81 0.58 0.8
multinom 0.61 0.69 0.33 0.6 0.38 0.39 0.39 0.8 0.61 0.66 0.61 0.59 0.8 0.59 0.79
ENet 0.59 0.67 0.33 0.57 0.35 0.36 0.36 0.79 0.59 0.66 0.59 0.56 0.79 0.56 0.78
Lightgbm 0.54 0.61 0.33 0.6 0.22 0.25 0.28 0.78 0.58 0.55 0.47 0.74 0.47 0.75
DT 0.54 0.61 0.33 0.62 0.22 0.26 0.29 0.78 0.65 0.57 0.47 0.63 0.47 0.75
KNN 0.59 0.67 0.33 0.55 0.34 0.36 0.36 0.79 0.56 0.65 0.56 0.55 0.77 0.55 0.79
stacking 0.63 0.69 0.33 0.57 0.38 0.41 0.43 0.83 0.6 0.62 0.6 0.57 0.75 0.57 0.8
MLP 0.57 0.65 0.33 0.52 0.3 0.32 0.32 0.78 0.54 0.56 0.54 0.53 0.73 0.53 0.77
SVM 0.55 0.63 0.33 0.46 0.25 0.29 0.3 0.79 0.48 0.53 0.48 0.49 0.69 0.49 0.76
ENet 0.51 0.61 0.33 0.45 0.21 0.24 0.24 0.76 0.46 0.52 0.46 0.46 0.68 0.46 0.75
multinom 0.51 0.61 0.33 0.45 0.21 0.24 0.24 0.76 0.45 0.52 0.45 0.46 0.68 0.46 0.75
RF 0.51 0.6 0.33 0.44 0.2 0.23 0.23 0.75 0.45 0.5 0.45 0.46 0.67 0.46 0.74
XGBOOST 0.51 0.6 0.33 0.44 0.2 0.23 0.23 0.76 0.44 0.5 0.44 0.46 0.66 0.46 0.74
Lightgbm 0.55 0.62 0.33 0.62 0.23 0.27 0.3 0.79 0.45 0.55 0.48 0.66 0.48 0.76
DT 0.53 0.59 0.33 0.58 0.19 0.22 0.27 0.78 0.63 0.56 0.45 0.59 0.45 0.74

| | | Training Set | Validation Set | *P*-value |
|---|---|---|---|---|
| Eyes | | 226 | 76 | |
| Gender (M:F) | | 71:42 | 26:12 | 0.412 |
| Age | | 9.58±8.93 | 8.84±7.38 | 0.554 |
| Z-AL | | 1.87±2.66 | 2.34±3.31 | 0.246 |
| Z-CCR | | 2.00±1.17 | 1.76±1.70 | 0.207 |
| Z-WTW | | 0.50±1.47 | 0.15±1.27 | 0.159 |
| CCT | | 547.31±50.78 | 542.61±43.18 | 0.555 |
| Cyl | | -1.77±1.01 | -1.82±0.98 | 0.731 |
| CECs | | 3282.52±431.19 | 3207.10±545.53 | 0.304 |
| IOP | | 14.83±3.67 | 13.92±2.98 | 0.126 |
| PO-1m BCVA | | 4.72±0.20 | 4.72±0.18 | 0.971 |
| Variants | DN(-Cys)&HI | 55 | 19 | 0.389 |
| | DN(Others) | 46 | 11 | |
| Terminal | N-terminal | 56 | 17 | 0.858 |
| | Middle Region | 32 | 11 | |
| | C-terminal | 13 | 3 | |
| Domain | cb EGF-like | 58 | 21 | 0.654 |
| | EGF-like | 13 | 3 | |
| | TGFBP | 9 | 3 | |
| | Hybrid | 8 | 1 | |
| | LTBP-like | 6 | 0 | |

**Supplementary Table 1.** Consistency test of the demographic ocular and genetic parameters of the training set and the validation set. Z-AL: Z-score for axial length; Z-CCR: Z-score of corneal radius of curvature; Z-WTW: Z-score for white to white; CCT: central corneal thickness; Cyl: corneal astigmatism; CECs: corneal endothelial cells; IOP: intraocular pressure; PO-1m BCVA: best corrected visual acuity at 1 month after surgery
